# H-shaped modifiers loaded mirror symmetric resonator based double negative metamaterial for multi-band wireless communications

**DOI:** 10.1038/s41598-023-43182-y

**Published:** 2023-09-24

**Authors:** Abdullah Al Mahfazur Rahman, Mohammad Tariqul Islam, Md. Moniruzzaman, Sharul Kamal Abdul Rahim, Mandeep Singh, Norbahiah Misran, Md. Shabiul Islam, Mohamed S. Soliman

**Affiliations:** 1 Department of Electrical, Electronic and Systems Engineering, Faculty of Engineering and Built Environment, Universiti Kebangsaan Malaysia, 43600 Bangi, Selangor Malaysia; 2 Department of Electrical and Electronics Engineering, College of Engineering and Technology, International University of Business Agriculture and Technology, Uttara, Dhaka, 1230 Bangladesh; 3 Wireless Communication Center, School of Electrical Engineering, Faculty of Engineering, Universiti Teknologi Malaysia, 81310 Johor Bahru, Malaysia; 4 Faculty of Engineering, Multimedia University, Cyberjaya, Malaysia; 5 Department of Electrical Engineering, College of Engineering, Taif University, P.O. Box 11099, Taif, 21944 Saudi Arabia

**Keywords:** Materials science, Nanoscience and technology, Physics

## Abstract

In this article, a unique metamaterial (MTM) structure is presented that exhibits four resonances of transmission coefficient (S_21_) that fall into S, X, and Ku bands. The MTM design is initiated on a Rogers (RT5880) substrate with an electrical dimension of 0.088 λ × 0.088 λ (λ is calculated at 3.424 GHz). The resonating patch contains four quartiles connected by a central metallic strip. The placement of each quartile is such that the whole resonator is mirror symmetric about the vertical axis. Two H-shaped modifiers connect two quartiles of each vertical half of the resonator. These H-shaped modifiers form the resonance cavity in its vicinity, and thus help significantly to orient the overall resonances of the proposed MTM at 3.424 GHz, 10 GHz, 14.816 GHz, and 16.848 GHz. The resonance phenomena are examined through equivalent circuit modeling and verified in Advanced Design Software (ADS). Metamaterial properties of the proposed MTM are extracted and it exhibits negative permittivity, permeability, and refractive index. The prototype of the MTM is fabricated and measurement is taken. The measured S_21_ shows a close similarity with the simulated result. Moreover, effective medium ratio (EMR) is calculated for the proposed MTM and a high EMR of 10.95 is obtained that expresses its compactness. This compact MTM with negative permittivity, permittivity, and refractive index can be important component for improving the performance of the miniaturized devices for multi-band wireless communication systems.

## Introduction

The latest development of wireless technologies expedited the applications of multiband frequency in a single microwave device. To meet future demand these devices must attain the miniaturization feature and as well as operation in multi-frequency ranges with high gain. There are artificial materials with a symmetrical homogeneous structure known as metamaterials, which can exhibit these properties together^1–3^. These symmetrical homogeneous structures of metamaterials are one of the prime developments in the designing and miniaturization of multiband microwave devices. These artificial metamaterials can exhibit many exclusive features that are not present in the established conventional materials^4–6^. One of them is negative permeability and permittivity at the same frequency. A metamaterial can display these unique properties depending on the structure’s orientation, geometry, and substrate materials. Nowadays, the research on metamaterials is one of the first growing research fields because of their small structure compared to the wavelength of the targeted electromagnetic wave^1,7–10^. The metamaterial-based system can be used effectively in different microwave applications such as sensing^1,11^ sound engineering, anomalous reflection, sub-wavelength focusing, and metallic cloaking^11,12^. Keshavarz et al*.*^13^ demonstrated a mechanism to detect skin cancer, where they used a metamaterial-based system in the terahertz frequency range. Using conventional reflect-array wide-band dual frequencies cannot be shared by the same aperture, which was possible by replacing it with a metasurface as explained by Nayeri et al*.*^14^ in 2018. In^15^ metasurface was used effectively in Holography and nanoprinting. In^16^ another ultra-compact metasurface was presented for polarization splitter–rotator application. A metamaterial-based active amplifier is presented in^17^, which can amplify and modify the magnitude of energy of the propagating spatial waves using digital coding. A metamaterial-based design to operate in the microwave frequency ranges is presented in^18^, which can cover wideband frequencies. In addition, this design of metamaterial can exhibit a high value of the effective medium ratio (EMR), negative permittivity, and refractive index close to zero. The metamaterial with the split ring is used in^19^ to achieve high EMR and negative permittivity. The proposed metamaterial of^19^ can cover frequencies of S, C, and X-bands. Misran et al*.*^20^ demonstrated the design of another split ring metamaterial that observed the impact of parameter variation with the help of thickness change and permittivity of the substrate material. To perform linear polarization Wang et al*.*^21^ presented a dual-band metamaterial-based converter. The model showed insensitive behavior to the polarization angle, which helped to reduce the RCS radar, imaging, and communication system^21^. Xie et al*.*^22^ proposed a Fabry–Perot antenna to reduce the RCS of radar, which used metamaterial in both transmitter and receiver surfaces, obtained high gain, and reduced the RCS of radar significantly.

Numerous research works have been conducted on metamaterial-based systems for multiband microwave applications. To facilitate the overall performance of the microwave system the performance of the antenna is very vital and can be evaluated by the bandwidth, gain, radiation efficiency, and size of the antenna. Bougoutaia et al*.*^23^ designed an antenna to attain high gain and wide bandwidth that used a dual ring metamaterial resonator in the antenna. The metamaterial resonator along with partial ground has helped the antenna to exhibit impedance-matching phenomena^23^. Van Yem et al*.*^24^ also improved the gain and bandwidth of the antenna by including a metamaterial structure on the ground plane of the antenna. A metamaterial-loaded mushroom-shaped antenna displayed circular polarization with high gain and wide bandwidth^25^. Along with high bandwidth, the efficiency of the antenna can also be improved by using metamaterial which is demonstrated in^26^ where the antenna is designed to attain the application-oriented frequency of 2.4 GHz. Pyo et al*.*^27^ improved the efficiency of the antenna-based transmission line model that used metamaterial in both central-fed and offset-fed antennas of the model. In^28^ the compactness of the antenna was achieved by the use of metamaterial in the design which reduced the size of the antenna substantially compared to the conventional antennas. To improve the antenna performance metamaterial-based split ring is presented in^29,30^. In^29^ a compact design of metamaterial was developed by tuning the split ring inductively which can provide negative permittivity and close to zero refractive index. To get these properties and improvement of antenna gain the model of ^30^ used a symmetrically shaped split ring with the coupled gap. Moreover, the metamaterial-based resonators can also be implemented in sensing applications. Using metamaterial in the structure of the sensor, the optical, electromagnetic, and mechanical properties of the sensors can be improved^31^.

Based on the above literature study, a double negative metamaterial is presented in this paper that has a high possibility of being used in future for the wireless communication applications like the performance improvement of antenna used in microwave communication systems and sensing devices. The unique features of the proposed MTM are: (i) it contains four quartiles placed in a 2 × 2 matrix where quartiles are connected at the center with a copper strip. The total structure is mirror symmetric about the vertical axis, which helps to reduce the cross-coupling effect between the array elements. (ii) The H-shaped modifiers are placed between two adjacent quartiles of the same column that help to improve the frequency response covering the low-frequency range to a high frequency. (iii) Resonances in different other frequencies can be selected by placing H-shaped modifiers at different other quartiles. (iii) Moreover, the H-shaped modifier also helps to improve the EMR value significantly, representing the compactness of the design. (iv) Additionally, the proposed MTM exhibits both permittivity and permeability negative in the vicinity of resonance frequencies of S_21_. Because of these unique features, the proposed double negative metamaterial can be utilized to develop a compact high-gain antenna. Moreover, the proposed MTM can also be used for the reduction of coupling effects in MIMO antennas. In addition to this, it can be used as a band-stop filter that can be used to hinder unwanted transmitted signals. Thus, the multiband resonance characteristics of this compact MTM make it suitable for use directly in various miniaturized microwave systems, or as a part of an antenna for performance enhancement. This MTM-loaded antenna can be employed in several applications such as in 5G communications, weather monitoring systems, amateur radio and VSAT systems as the designed frequencies of the proposed MTM fall in the S, X, and Ku bands. The rest of the paper is organized as follows. The design of the MTM unit cell is presented in section two. This section also includes step-by-step design procedures, electromagnetic field and current analysis, and effective parameter extraction methods. The equivalent circuit model is demonstrated in section three. In section four, the result is analyzed, which includes simulation and measurement results, analysis of effective parameters, effects of substrate change of the designed MTM, comparison of the unit cell of the proposed MTM with its different arrays, impact of oblique incident angle change of the proposed MTM, a comparative study of the effects of H-shaped modifiers at different positions, Far-field analysis of the proposed MTM and comparison of proposed model with relevant works. Finally, a conclusion is drawn in section five that includes the major outcome of this work.

## Design and analysis of the simulation model of the unit cell of the proposed model

### Development of the structure of the unit cell of the proposed model

**Figure 1:**
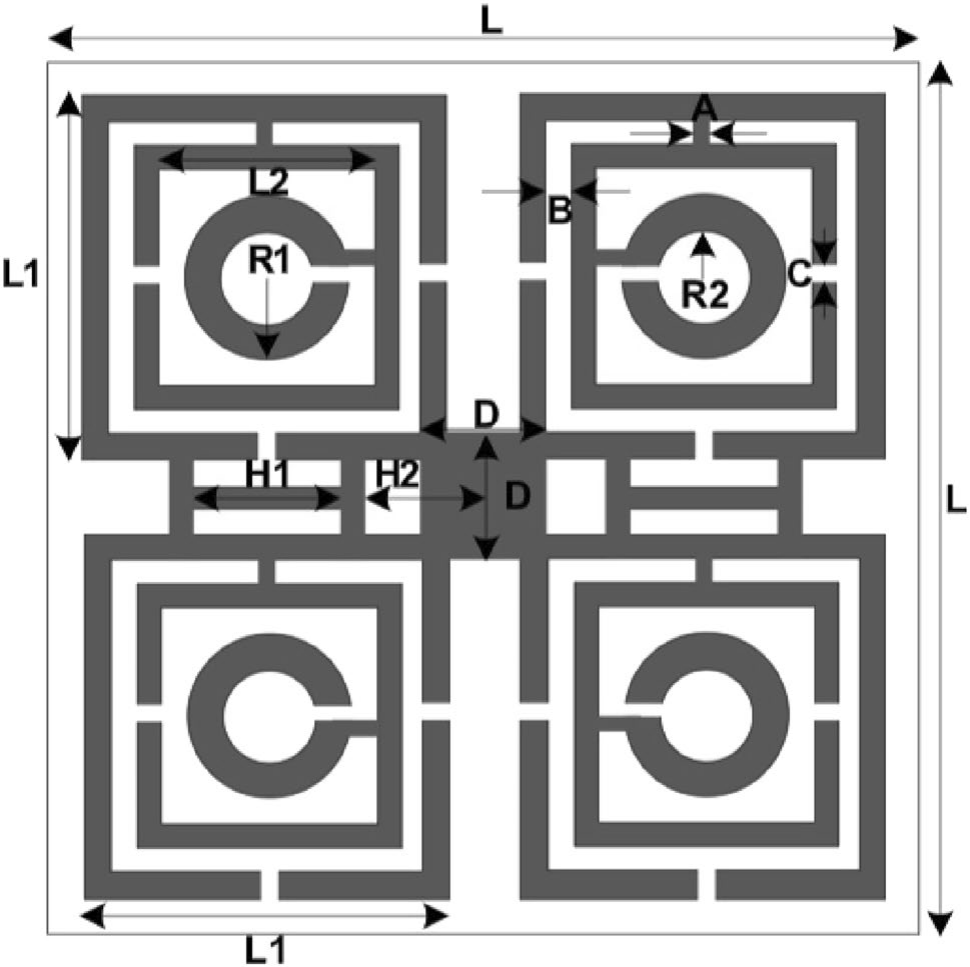
Dimension of the unit cell of the proposed model.

The schematic diagram of the unit cell of the proposed model is given in Fig. 1. As the model is targeting wide ranges of frequency bands, Rogers RT5880 is suitable for the application. The motivation behind selecting this material as the substrate because it can provide minimum moisture absorption and minimum electrical losses. At the same time, over a wide range of frequency bands, it can exhibit identical electric properties. For the proposed model the thickness of the substrate is selected to 1.57 mm with dielectric constants of 2.2 and loss tangent of 0.0004. To make the dimension of the unit cell small compared to the wavelength, an 8 × 8 mm^2^ substrate is chosen for the unit cell. The frequency range for the system is considered 2 to 18 GHz. This material is used as the substrate of the designed resonator patch and on top of this conducting metal strip is used with a thickness of 0.035 mm. The patch is constructed by combining four identical segments. Each quartile has two square-shaped split ring resonators (SRR) and one circular split ring resonator (CSRR) that are connected by three metal strips. Then these four segments are connected by a central rectangular conducting metal strip. The upper two quartiles are connected with the lower quartiles by two H-shaped modifiers. The dimension of the unit cells is provided in Fig. 1. The various segments are presented in Table 1.

**Table 1:** Dimensions of the proposed model.

Part	L	L1	R1	R2	A	B	C	D	H1	H2
Dimension (mm)	8.0	3.5	0.7	0.40	0.20	0.25	0.20	1.10	1.30	0.65

**Figure 2:**
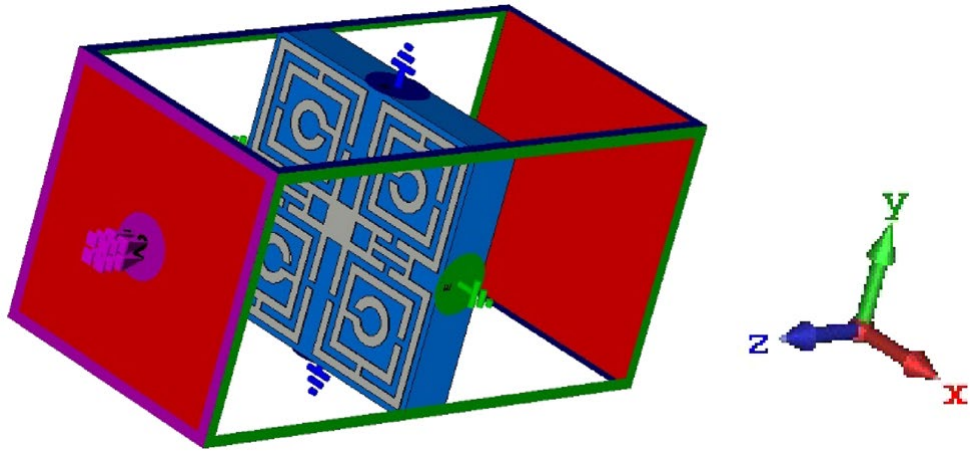
Placement of the simulation model (CST studio).

To prepare the simulation setup and evaluate the properties of the proposed model of metamaterial, its unit cell is sited in the middle of positive and negative Z direction of the wave-guide ports of the CST Studio Suite 2019 simulator is shown in Fig. 2. For the simulation, the E field is set in the X and H field is set in Y direction.

### Steps towards the formation of the proposed model

**Figure 3:**
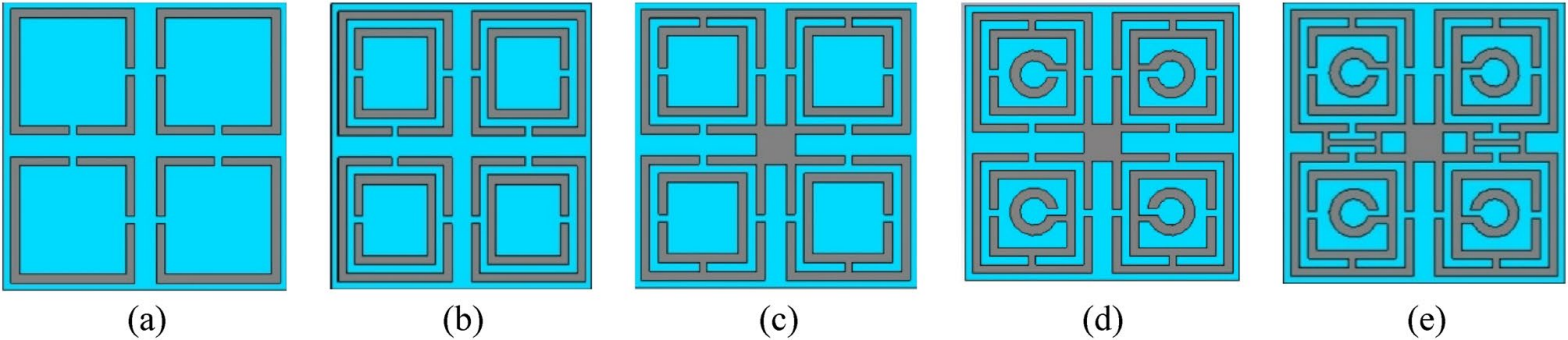
The successive advancement of the unit cell of proposed model as: (**a**) Design step 1, (**b**) Design step 2, (**c**) Design step 3, (**d**) Design step 4, and (**e**) Proposed model.

**Figure 4:**
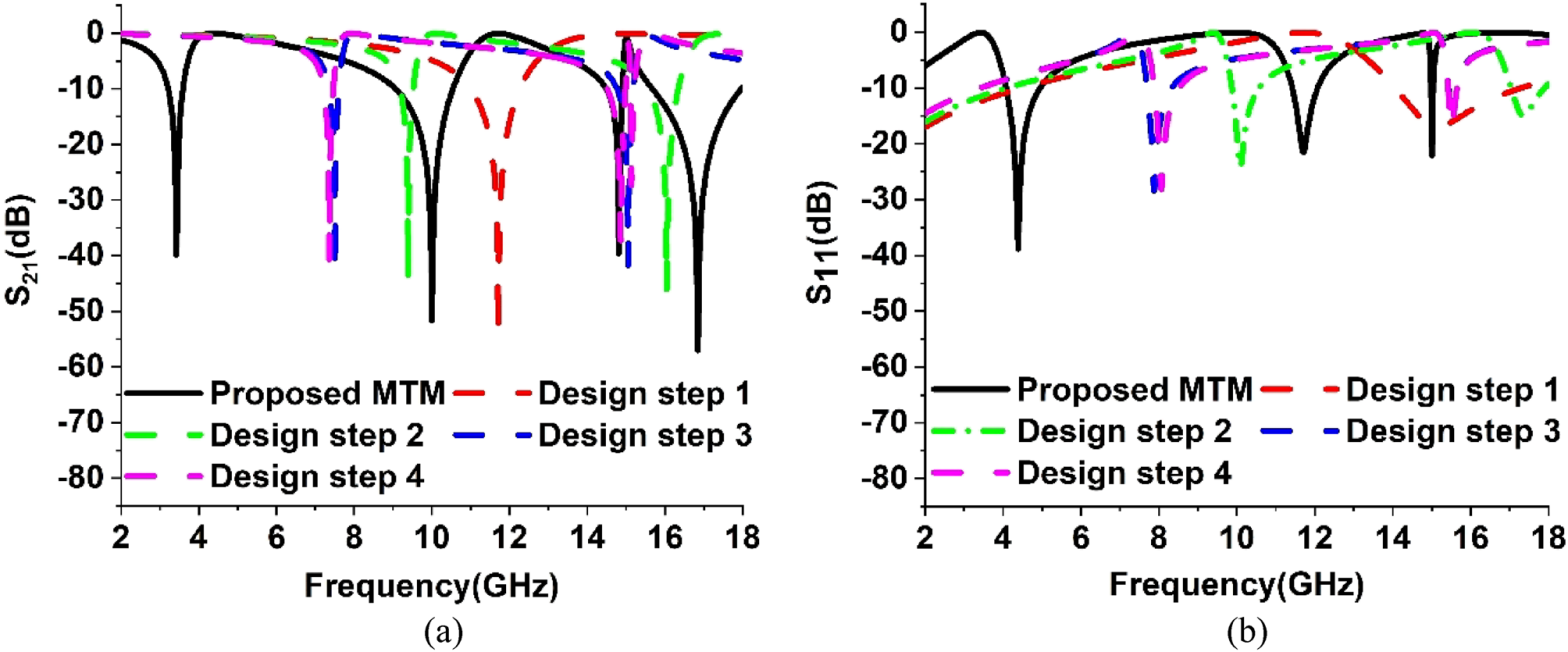
Scattering parameters: (**a**) S_21_, and (**b**) S_11_ for systematic development of the unit cell.

The design of the unit cell of the proposed model is developed with the help of a step-by-step modification process. The design steps are presented in Fig. 3. In each step, the response of the transmission coefficients (S_21_) and (S_11_) are recorded at their corresponding resonance frequencies. The resonance frequencies and bandwidths of S_21_ and S_11_ for different design steps are offered in Tables 2 and 3 correspondingly. The responses of S_21_ and S_11_ for these steps are plotted in Fig. 4a,b respectively. In Design step 1, four square-shaped identical split ring resonators are placed on each quartile of the substrate. To make the configuration axis symmetric two split gaps are created in the inner horizontal and vertical portions of each ring. This design attained the resonance of S_21_ at 11.712 GHz with a bandwidth of 1.184 GHz and S11 at 15.171 GHz with a bandwidth of 3.184 GHz. as shown in Fig. 4a,b respectively. In Design step 2, another split ring is added inside each split ring of Design Step 1. The split gaps are created in this design in the opposite direction of the vertical portion gaps of split rings of Design step 1. Similar to Design step 1, in this design, because of the influence of mutual inductance created by two split rings, the first resonance point of S_21_ shifted to 9.42 GHz and S_11_ to 10.099 GHz as shown in Fig. 4a,b respectively. An additional resonance of S_21_ is achieved at 16.084 GHz and S_11_ at 17.336 GHz due to the insertion of an additional split ring in each quartile of the substrate of this design. In Design step 3, four outer split rings of Design step 2 are connected at the center by a rectangular-shaped metallic strip. In this design, the two split rings of each quartile are also joined together with the help of a metal strip located at the opposite side of the horizontal split gap of a split ring of Design step 1. For the development of this design, the two resonances of S_21_ shifted on the left side at 7.344 GHz and 15.072 GHz respectively. On the other hand, the resonances of S_11_ shifted at 7.88 GHz and 15.537 GHz correspondingly. By adding a circular-shaped metallic split ring inside the split rings of Design step 3, Design step 4 is developed. Because of this Design step, two resonances of S_21_ of Design step 3 are shifted at 7.36 GHz and 15.104 GHz respectively. In addition, it generated a new resonance of S_21_ at 14.81 GHz. For this Design step, the first resonance of S_11_ of Design step 3 has shifted to 8.064 GHz whereas the second resonance is unaffected by this design. It has been noticed that up to Design step 4, the model could cover the resonance frequencies of S_21_ between 7.36 and 15.104 GHz and S_11_ between 8.064 and 15.501 GHz. Therefore, to cover the lower and higher resonances of S_21_ the proposed model is developed from the modifications of Design step 4. In this model, two horizontal H-shaped modifiers are placed between two upper quartiles and bottom quartiles of design step 4. These two metal H-shaped modifiers support attaining three new resonances of S_21_ at 3.424 GHz, 10 GHz, and 16.848 GHz along with the resonance at 14.816 GHz of the previous Design step. The proposed model also provided the resonances of S_11_ at 4.3858 GHz, 11.712 GHz, and 15.008 GHz.

**Table 2:** The resonance frequencies and their corresponding bandwidths of S_21_ for systematic development of the unit cell.

Design	Resonance frequency (GHz)	Bandwidth (GHz)
Design step 1	11.712	1.184
Design step 2	9.42 and 16.084	0.341 and 0.772
Design step 3	7.344 and 15.072	0.255 and 0.43
Design step 4	7.36, 14.816 and 15.104	0.2437, 0.365 and 0.96
Proposed Model	3.424,10,14.816 and 16.848	0.374 and 1.468 0.365 and 2.288

**Table 3:** The resonance frequencies and their corresponding bandwidths of S_11_ for systematic development of the unit cell.

Design	Resonance frequency (GHz)	Bandwidth (GHz)
Design step 1	15.171	3.184
Design step 2	10.099 and 17.336	0.6887 and 0.841
Design step 3	7.88 and 15.501	0.623 and 0.236
Design step 4	8.064 and 15.501	0.6311 and 0.236
Proposed Model	4.3858, 11.712 and 15.008	0.8677, 0.629 and 0.055

### Electric field, magnetic field, and surface current analysis of proposed model

**Figure 5:**
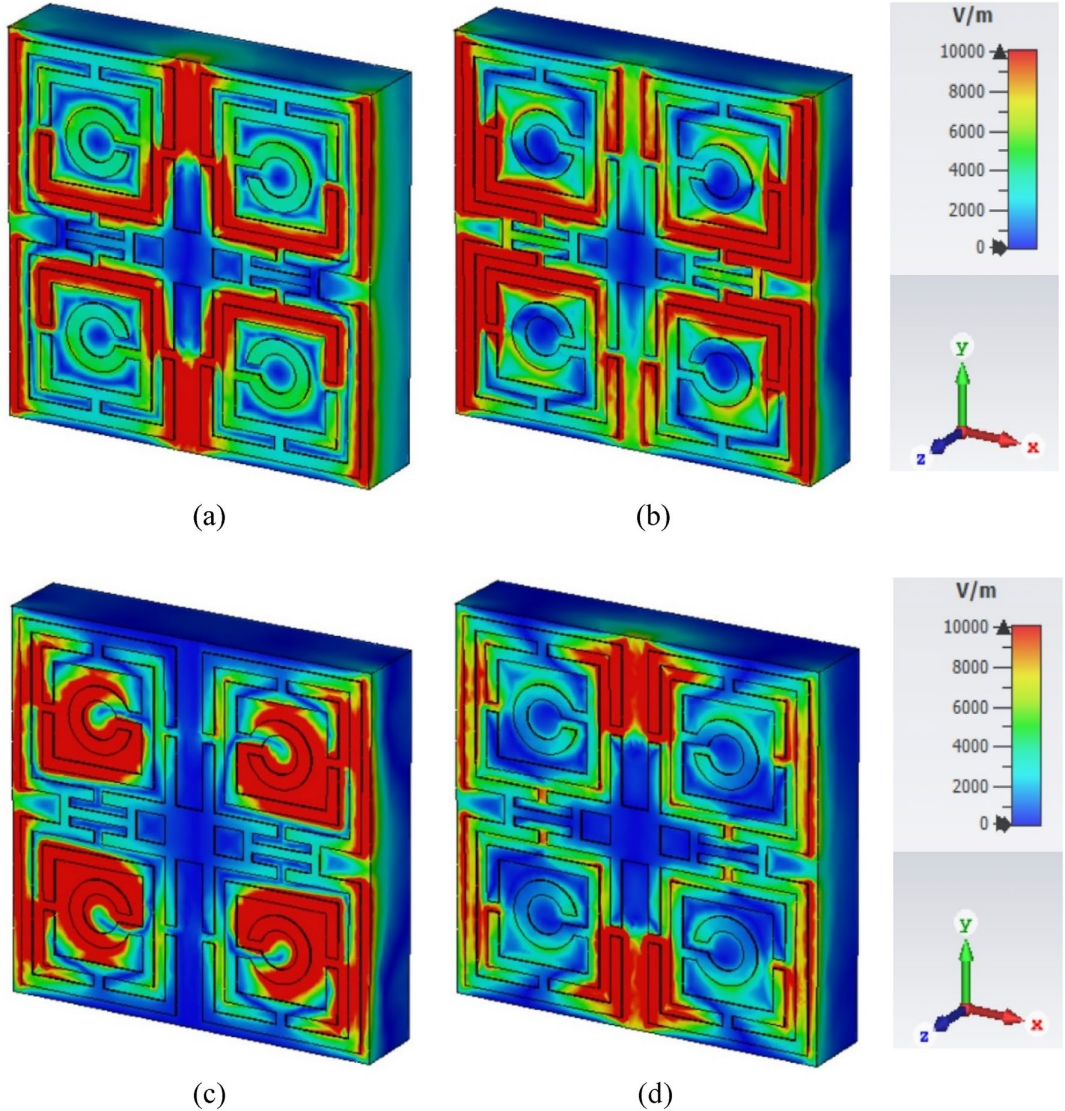
Influence of electric field for: (**a**) 3.424 GHz, (**b**) 10 GHz, (**c**) 14.816, and (**d**) 16.848 GHz.

**Figure 6:**
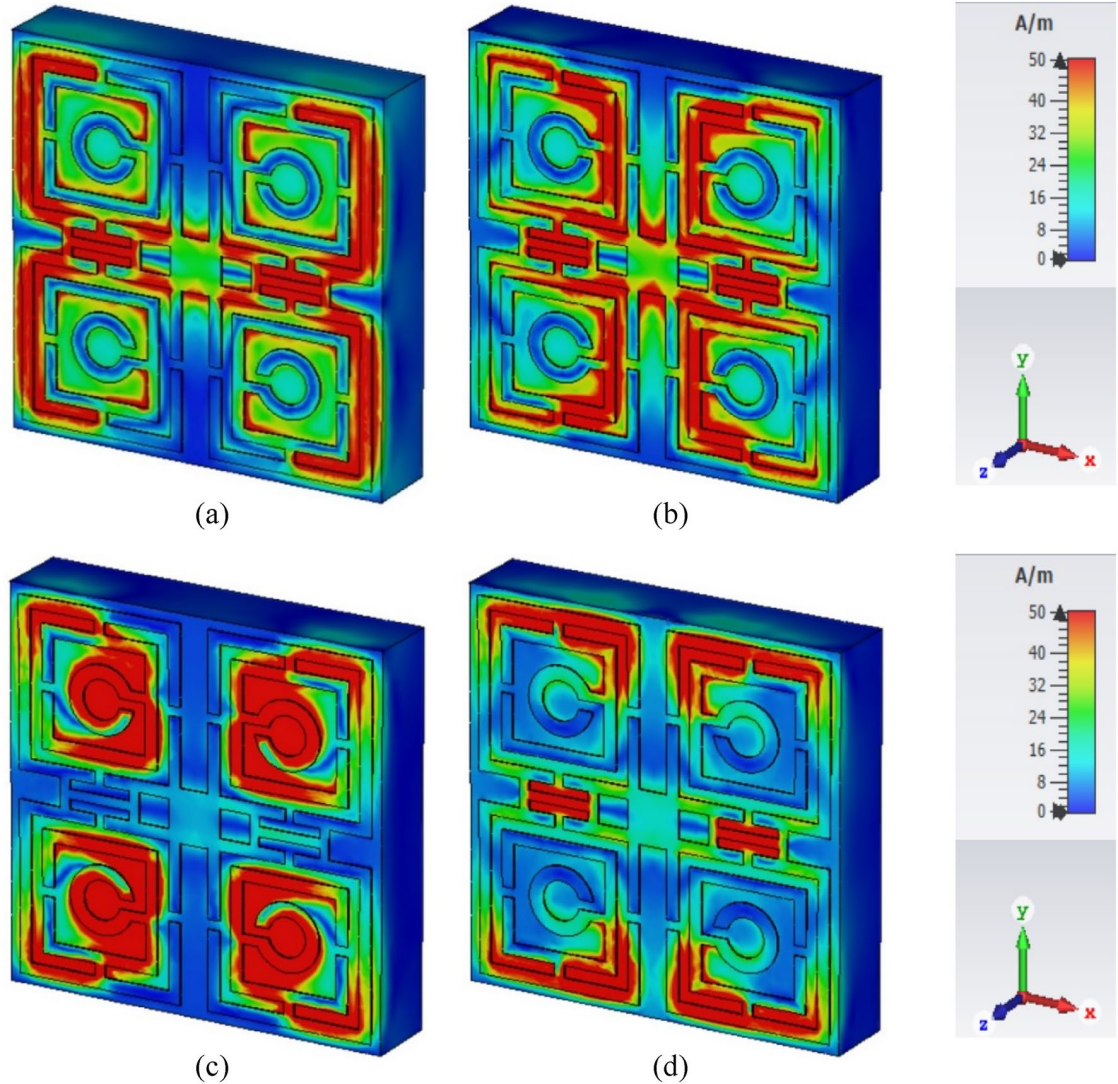
Influence of magnetic field for: (**a**) 3.424 GHz, (**b**) 10 GHz, (**c**) 14.816, and (**d**) 16.848 GHz.

**Figure 7:**
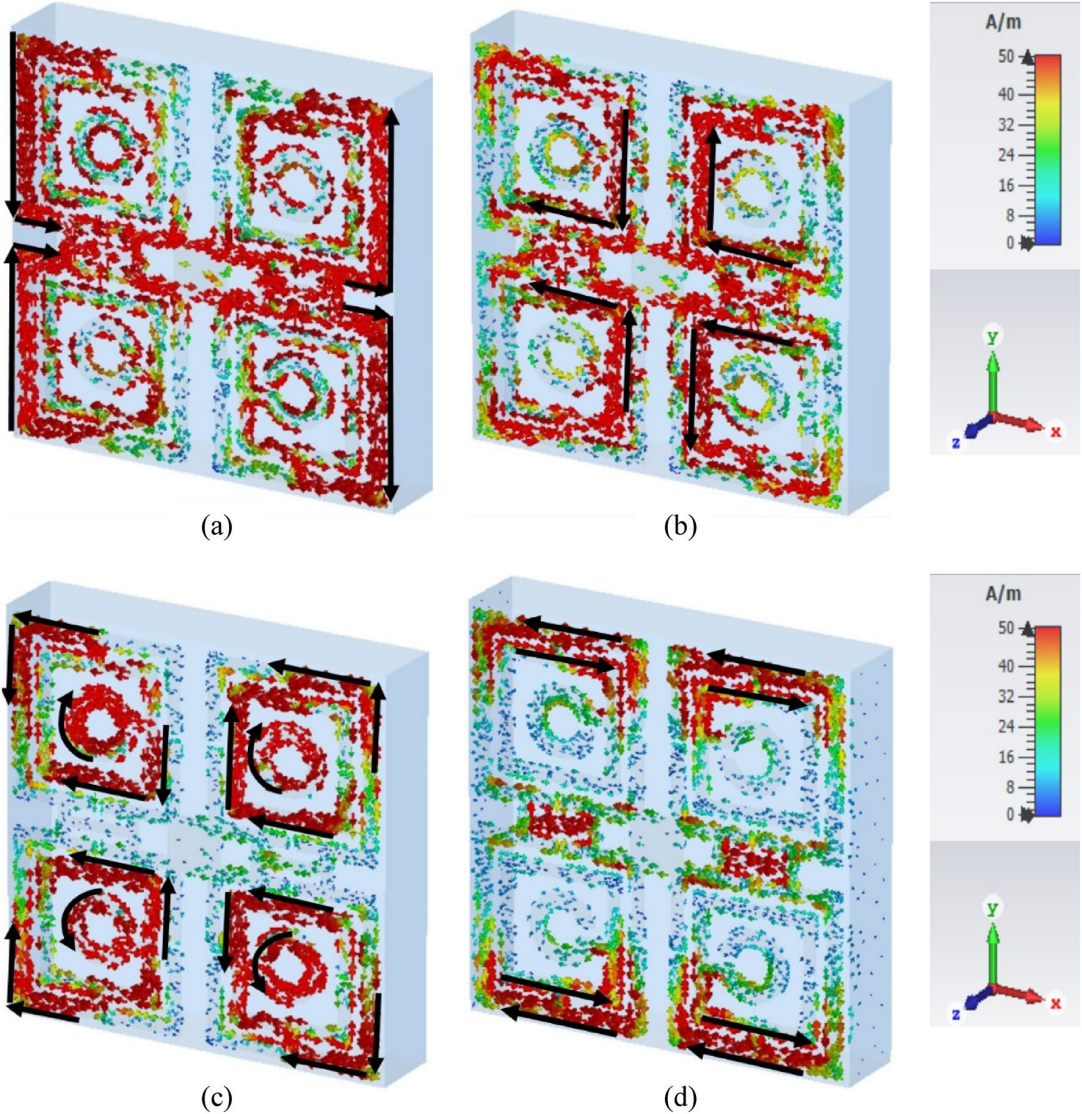
Influence of surface current for: (**a**) 3.424 GHz, (**b**) 10 GHz, (**c**) 14.816, and (**d**) 16.848 GHz.

The influence of the electric field, magnetic field, and surface current on the unit cell of the proposed MTM can be observed in Figs. 5, 6, and 7 respectively for the resonance frequencies of 3.424 GHz, 10 GHz, 14.816, and 16.848 GHz. There is a correlation among these parameters, which can be expressed mathematically using Maxwell’s equations as explained in^32^. At 3.424 GHz, a strong electric field appears near the outer vertical segment of the first rectangular ring and the inner half of the second split ring of each quartile of the resonator as shown in Fig. 5a. The electric field distribution has changed at 10 GHz, where it is reduced in the inner vertical parts of the first and second rectangular rings and increased significantly in the outer vertical parts of the first and second rectangular rings of each quartile as exposed in Fig. 5b. At 14.816 GHz, the electric field strength is high near the outer vertical segment of the first and second rectangular rings, the inner horizontal segment of the second rectangular rings, and the third circular ring as depicted in Fig. 5c. At 16.848 GHz, the electric field increased significantly in the upper half of the inner vertical region of the outer rectangular ring of each quartile as shown in Fig. 5d. The magnetic field distribution became very strong in the outer region of the first rectangular ring of each quartile and the H-shaped modifiers at 3.424 GHz which is presented in Fig. 6a. A substantial magnetic field appeared in the inner part of the second ring of each quartile and the H-shaped modifier segment but disappeared from most of the part of the first outer ring at 10 GHz frequency, as shown in Fig. 6b. However, at 14.816 GHz, the magnetic field disappeared from the H-shaped modifiers whereas it became very strong at circular split rings and most of the parts of the second rings as shown in Fig. 6c. At 16.848 GHz, a strong magnetic field appeared in the outer horizontal segments of the first and second rectangular rings of each quartile and the H-shaped modifiers of the resonator as shown in Fig. 6d. The impact of the magnetic field can also be justified through the image of the surface current as presented in Fig. 7. As with the magnetic field, the surface current became very strong in the outer region of the first rectangular ring of each quartile, and the H-shaped modifiers at 3.424 GHz that are presented in Fig. 7a. At 10 GHz frequency, the surface current is very strong in in the inner part of the second ring of each quartile and the H-shaped modifier segment. Surface current is also very large at the horizontal H-shaped modifiers as shown in Fig. 7b. At 14.81 GHz the surface current of the H-shaped modifiers becomes very small whereas it becomes very high at the circular split ring and most of the part of the second ring of each quartile as shown in Fig. 7c. At 16.848 GHz, the high current distribution is observed in the outer horizontal segments of the first and second rectangular rings of each quartile and the middle of H-shaped modifiers of the resonator as shown in Fig. 7b.

### Effective parameters extraction procedure

The effective parameters of the proposed model can be extracted by the Nicolson–Ross–Weir (NRW) method that is explained in^33,34^. To extract permittivity ε_r_, permeability μ_r_, normalized impedance Z, and refractive index η_r_ the values of scattering parameters S_21_ and S_11_ are used from the CST simulation. As explained in Ref^29,33,35,36^ the effective permittivity, ε_r_ and permeability, μ_r_ can be calculated using the following Eqns.:

1 $${\upvarepsilon }_{\text{r}}=\frac{\text{c}}{\text{j} {\pi}fd}\times \frac{(1-{\text{S}}_{21}-{\text{S}}_{11})}{(1+{\text{S}}_{21}+{\text{S}}_{11})}$$

and,

2 $${\upmu }_{\text{r}}=\frac{\text{c}}{\text{j}{\pi} fd}\times \frac{(1-{\text{S}}_{21}+{\text{S}}_{11})}{(1+{\text{S}}_{21}-{\text{S}}_{11})}$$

The normalized impedance can be calculate as:

3 $$\text{Z}=\sqrt{\frac{{\upmu }_{\text{r}}}{{\upvarepsilon }_{\text{r}}}}$$

The effective refractive index η_r_ is calculated from S_11_ and S_21_ using direct refractive strategy,

4 $${\upeta }_{{\text{r}}} = \frac{{\text{c}}}{{{\text{j}{\pi} fd}}} \times \sqrt {\frac{{\left( {{\text{S}}_{21} - 1} \right)^{2} - \left( {{\text{S}}_{11} } \right)^{2} }}{{\left( {{\text{S}}_{21} + 1} \right)^{2} - \left( {{\text{S}}_{11} } \right)^{2} }}}$$

where c represents the velocity of light, f is the frequency of the signal and d is the thickness of the substrate.

A MATLAB code is used where equations from (1) to (4) are used to calculate the permittivity, permeability, and normalized impedance, and refractive index of the proposed MTM. The effective parameters can also be extracted from CST simulation which uses Drude–Lorentz model^29^ for S-parameters identification and built in post processing module for effective parameters.

## Equivalent circuit modeling of the proposed MTM unit cell

**Figure 8:**
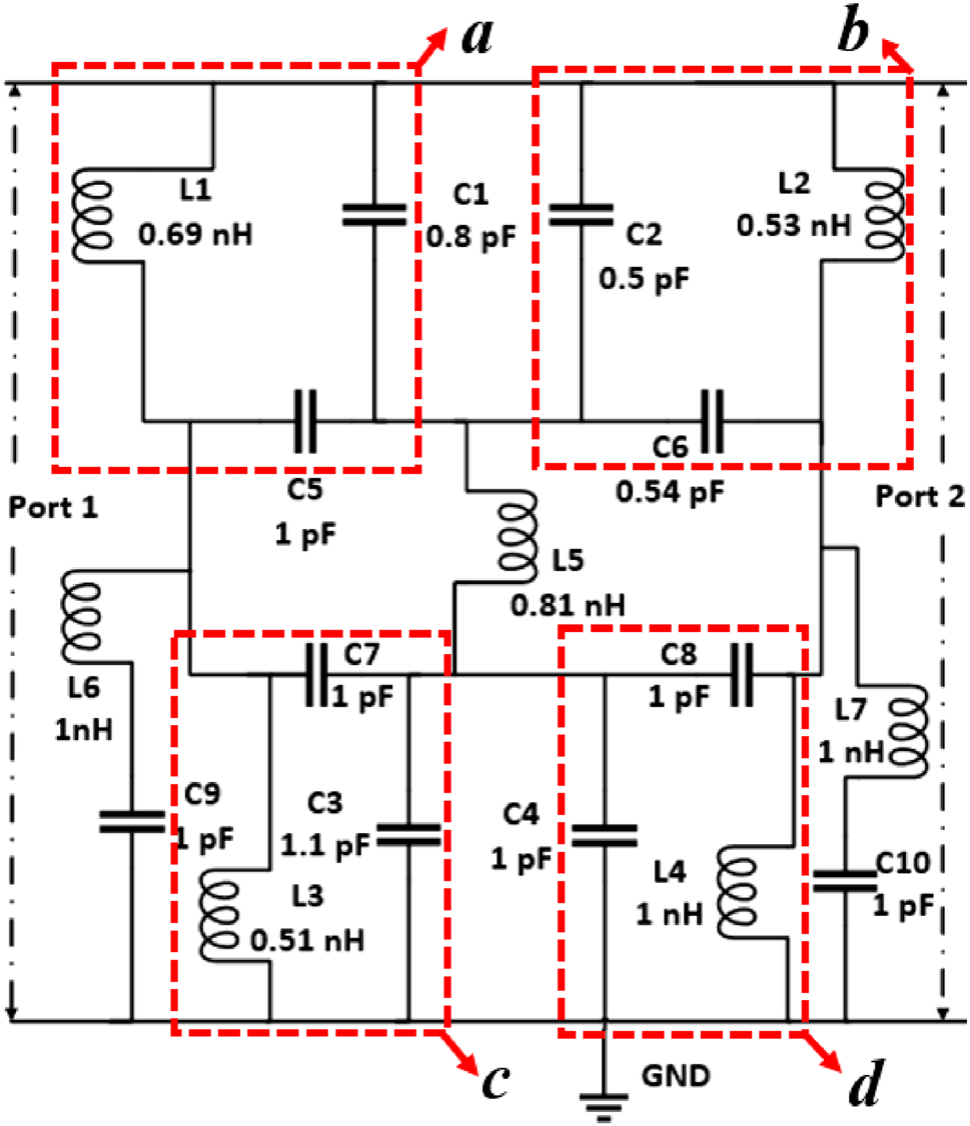
Equivalent circuit of the proposed model.

**Figure 9:**
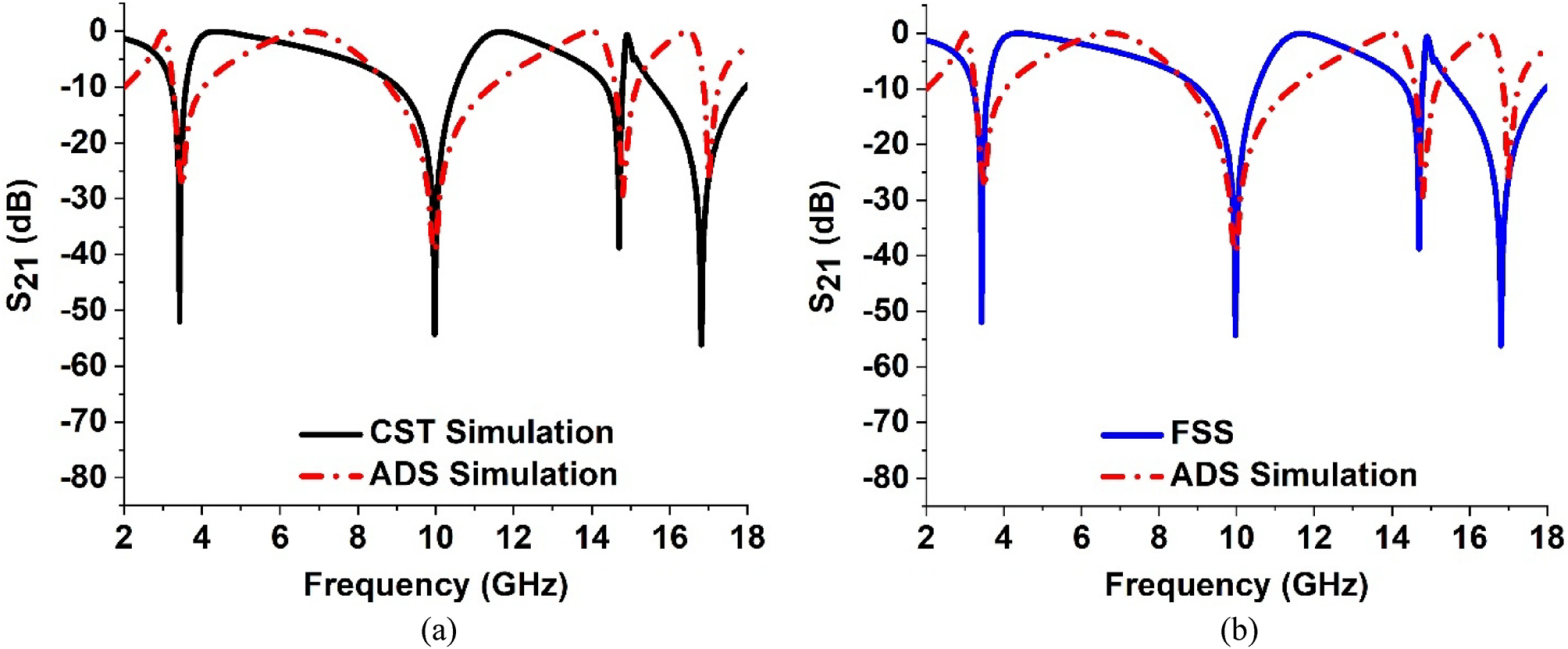
S_21_ spectra for the simulation of: (**a**) unit cell in CST and equivalent circuit of the proposed model in ADS, and (**b**) Frequency selective surface (FSS) and equivalent circuit of the proposed model in ADS.

For understanding the resonance phenomena, the equivalent circuit of the proposed MTM is modeled considering the inductive effect of the metallic strips and the capacitive effect of the split gaps. The equivalent circuit for the proposed MTM unit cell is depicted in Fig. 8. Since the unit cell contains four quartiles, in the equivalent circuit each quartile is represented by corresponding inductance and capacitances. In Fig. 8, these quartiles are represented by the circuit segments labeled as a, b, c and d. These four quartiles are connected to each other by an inductive metallic strip whose equivalency is presented in the circuit by inductance L5. The effects of the H-shaped modifiers that help to modify the resonances are presented by two series-connected LC circuits, one is combined with an inductance and capacitance pair, L6 and C9, and another is formed with L7 and C10. The circuit is modeled in Advanced Design Software (ADS) considering the initial values for each inductor of 1 nH and each capacitor of 1 pF. The two ports are connected at two ends of the circuit as shown in Fig. 8 and the S-parameter is noticed. The values of the components are tuned by using the tuning module of ADS so that the equivalent circuit provides the transmission coefficient (S_21_) as like the S_21_ of the 3D modeling in the CST. Thus, the circuit components are finalized when both the transmission coefficients show close similarity. The transmission coefficient (S_21_) plot obtained from the equivalent circuit is presented in Fig. 9a. By scrutinizing Fig. 9a, it is observed that the resonances obtained around frequencies of 3.42 GHz, 10 GHz, and 14.82 GHz are nearly in the same frequencies, comparing the graph obtained from the ADS circuit with the graph obtained from the CST simulation. On the other hand, resonances around 16.85 GHz show a deviation in frequency and bandwidth. The frequency deviation is nearly 0.9% though the bandwidth deviation is quite large. This discrepancy in bandwidth can be minimized by more precisely tuning the circuit components and considering the resistive effects of the inductors. For the simplification of the circuit, resistive effects are ignored. Moreover, the 3D design of the MTM unit cell in CST considers the inductive effect in distributed form. Whereas, in the circuit simulation we consider this effect in lumped form. Additionally, the effects of parasitic capacitances are omitted in the circuit model of Fig. 8. Despite all these effects, the equivalent circuit presented in Fig. 8 well represents the proposed MTM unit cell, which is obvious from the plots expressed in Fig. 9a. In addition, the ADS simulation result is also compared with the frequency selective surface (FSS) simulation in CST which is presented in Fig. 9b. To replicate the behavior of FSS, a array of 10 × 10 of unit cells of the proposed MTM are constructed in CST and simulation is conducted. The simulation result of FSS is also closely matched with the equivalent circuit result of the proposed MTM in ADS, which indicates that the proposed circuit model can perform well at the continuous region on the FSS.

## Result analysis

### Analysis of simulation and measurement results

**Figure 10:**
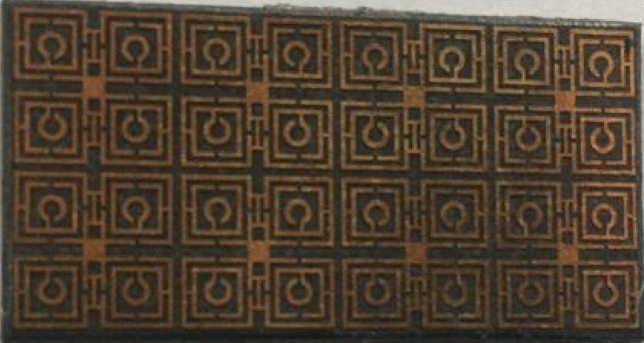
Fabricated array of the proposed model.

**Figure 11:**
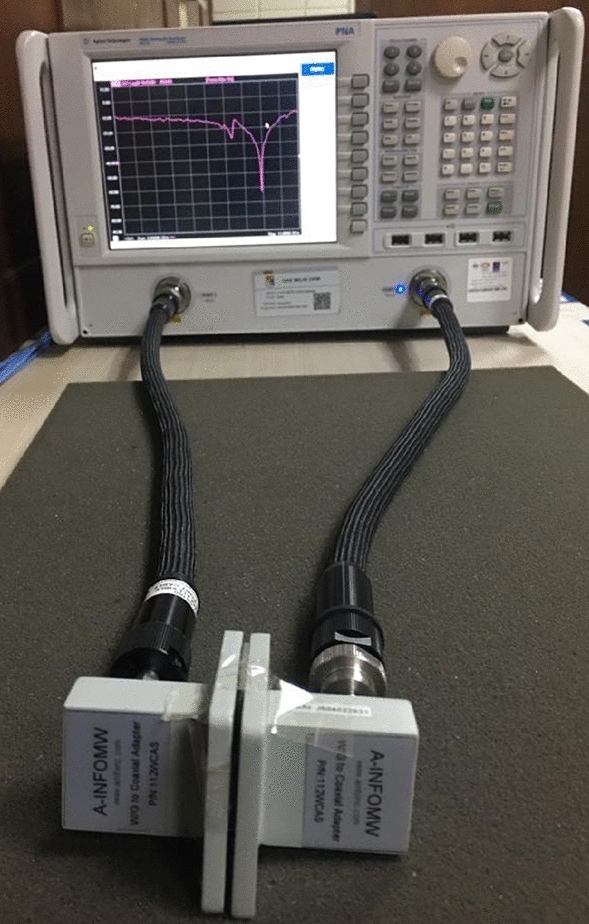
Experimental setup of array of the proposed model with vector network analyzed.

**Figure 12:**
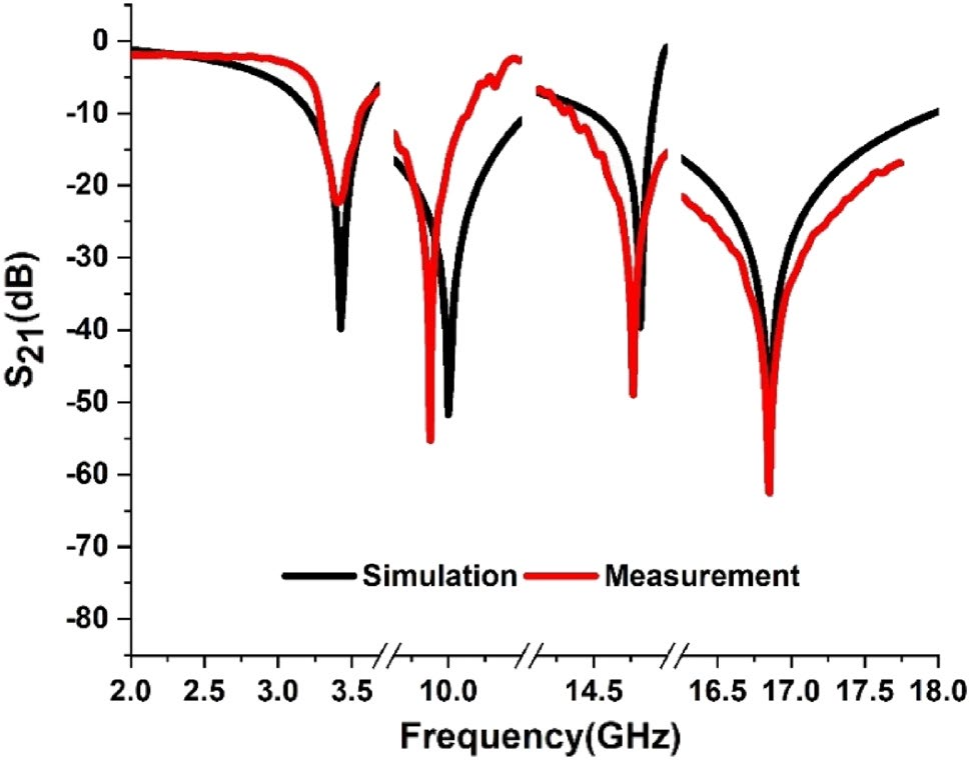
Simulation and Measurement results of S_21_.

The array of the proposed metamaterial-based model is given in Fig. 10. The prototype of the array is fabricated with the unit cell dimension of 8 × 8 mm^2^ for the measurement purpose. The performance of the prototype is analyzed using Agilent N5227A, Vector Network Analyzer (VNA) for determining the scattering parameters of the proposed unit cell. The vector network analyzer is calibrated with the help of electronic calibration kit (model: N4694-60001). The measurement setup is given in Fig. 11. The data recorded for S_21_ from the measurement is compared with the simulation results of CST, which is depicted in Fig. 12. From the measurement, the resonances of S_21_ are found at 3.4 GHz, 9.878 GHz, 14.76 GHz, and 16.849 GHz respectively whereas the simulation results provided the corresponding resonances of S_21_ at 3.424 GHz, 10 GHz, 14.816 GHz, and 16.848 GHz respectively that are plotted together in Fig. 12. After analyzing these two sets of data it is found that, from the simulation results there are frequency deviations of 0.7%, 1.22%, and 0.38% in the first three resonances of S_21_ respectively, whereas the fourth resonance frequency matches with the simulation result. The measurement data provides negative peak values of S_21_ of − 22.27 dB, − 55.26 dB, − 51.65 dB, and − 62.21 dB for the corresponding resonance frequencies whereas the simulation results provided the negative peaks of S_21_ of − 38.67 dB, − 48.36 dB, − 39.51 dB and − 52.93 dB for the corresponding resonance frequencies. The deviations of negative peak values of S_21_ from the simulation results are 42.41%, 14.26%, 30.72%, and 17.53%, respectively at the above mentioned frequencies of investigatoin.

From the analyses, it can be commented that the deviation of resonance frequencies from the simulation results to the measurement results are very negligible which implies that the resonance frequencies of the simulation are matched very closely with the measurement. However, there are disparities in the negative peak values of S_21_ between measurement results. A few aspects of the prototype design and measurement environment may be responsible for these inconsistencies. To be more specific, construction errors due to very tiny metal strips with miniaturization of slots, and calibration errors of the VNA may have occurred. However, the overall measurement response can be considered to have a significant level of matching between the simulation results, which can be observed in Fig. 12. The model performed very well in the high-frequency ranges. Irrespective of these inconsistencies, the deviation is not severe, and the experimental result exhibits similar behavior within the S, X, and Ku bands.

### Analysis of effective parameters

**Figure 13:**
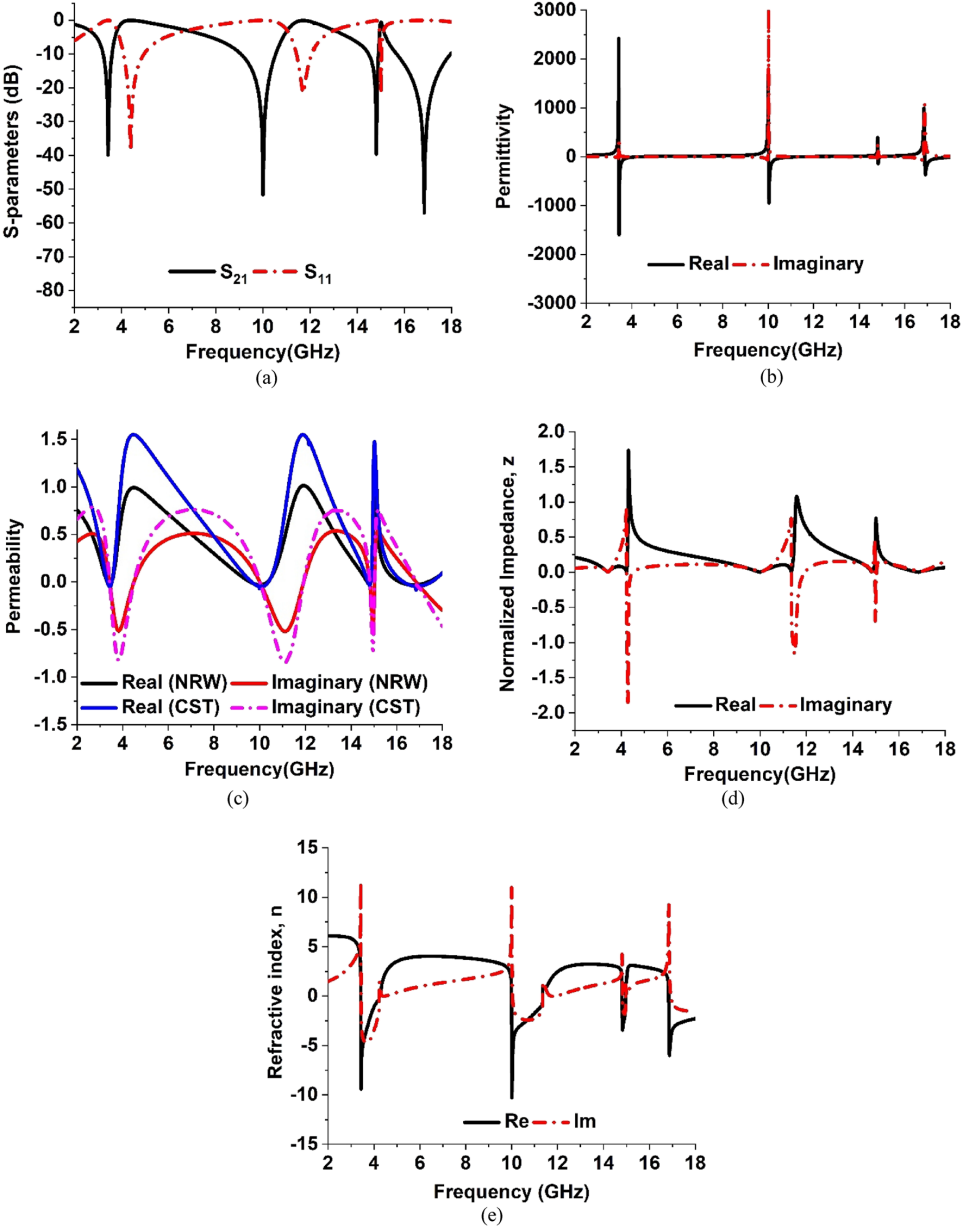
The simulated responses of parameters of the proposed model. (**a**) S_21_ and S_11_, (**b**) Permittivity ε, (**c**) Permeability μ, (**d**) Normalized Impedance z, and (**e**) Refractive Index n.

The S-parameters of the proposed MTM are presented in Fig. 13a. The values of permittivity and permeability are extracted using the Eqs. (1) and (2) respectively which require the values of S_21_ and S_11_. The permittivity and permeability plots are presented in Fig. 13b,c respectively. Table 4 summarizes the frequency ranges of the permittivity and permeability, normalized impedance, and refractive index of the proposed model near the resonance frequencies. As explained in^37^, the relation between permittivity and plasma frequency is established by the following Eq. (5):

5 $$\upvarepsilon =1-\frac{{{ \upomega }}_{\text{p}}^{2}}{{{ \upomega}}^{2}}$$

where ω represents the frequency of the incident EM wave and the plasma frequency is represented by ω_p_. When the frequency of the EM wave is less than the plasma frequency the permittivity becomes negative. It becomes zero if the frequency of the EM wave is equal to the plasma frequency.

**Table 4:** Effective parameters with frequency ranges of the proposed model.

Parameters	Frequency ranges in GHz near the resonance frequencies	Remarks
Permittivity, ε	3.433–4.193, 10.013–11.434, 14.828–14.984, 16.886–18.00	ε $<$ 0
Permeability, μ	3.329–3.531, 9.65–10.387, 14.744–14.87, 16.313–17.527	μ < 0
Normalized impedance, z	3.421–4.211, 10.15–11.314, 14.789–14.951	z ⁓ 0
Refractive index, n	3.4265–4.231, 9.998–11.351, 14.811–14.968, 16.84–17.996	n < 0

By observing the real part of the permittivity, ε of the proposed model, it is realized that permittivity undergoes transition from positive peak having magnitude of 2381 to negative peak of magnitude − 1596 at 3.424 GHz, from 1684 to − 945 at 10 GHz, and from 992 to –67 at 16.84 GHz which are significantly large. On the other hand, the real part of the permeability shows negative values of − 0.04827 at 3.456 GHz, − 0.070934 at 10 GHz, − 0.039874 at 14.816 GHz and − 0.085298 at 16.848 GHz.

From Table 4 it is observed that both permittivity and permeability are negative in the frequency ranges of 3.433–3.5131 GHz, 10.013–10.387 GHz, 14.828–14.87 GHz, and 16.886–17.527 GHz. The double negative properties of the metamaterials can be achieved using a split-ring resonator (SRR) that exhibits negative values of the permeability and micro-strip metallic lines or micro-structured array of wires to obtain negative permittivity that is followed by the original work of J. B Pendry et al. The works presented in Refs.^38–40^ are some examples that follow these principles to obtain the double negative properties of the metamaterial. Using of micro-structured array of wires and SRR helps to tune the magnetic and electric resonance separately and thus, the magnitude of the permittivity and permeability can be controlled robustly through structural design. On the other hand, only SRR-based double negative metamaterials are also exercised in Refs.^41–44^ in which complementary split ring resonators are used to obtain the double negative characteristics. In the present work, a one-sided design approach has been followed. Since both electrical and magnetic resonances are required to obtain both negative permittivity and permeability simultaneously, the proposed MTM is so designed that each quartile of this design contains three interconnected complementary split rings. Moreover, the quartiles are so arranged that they are mirror images of one another. Due to this structural design, an antiparallel surface current is flowing through various segments when the MTM is excited with electromagnetic waves. The analysis of surface current distribution at 3.424 GHz reveals that strong anti-parallel currents flow through the vertical arms of the outer split rings of resonator quartiles as shown in Fig. 7a. Compared to this, the inner arms of this ring contain almost zero currents. Thus, the magnetic dipole is formed between two vertical sides of the outer ring that contributes to magnetic oscillation with a result of shifting the permeability values from positive to negative values. On the other hand, a strong electric field concentrated near the innermost arms of the outer ring contributes to the electrical resonance and thus it assists in the transition of permittivity between positive and negative values. Similarly, the anti-parallel currents flown through various segments and rings of MTM at 10 GHz, 14.816, and 16.848 GHz as shown in Fig. 7b–d respectively triggers the magnetic resonances. Thus, magnetic as well as electric field distribution in different segments of the MTM contributes to double negative characteristics of the permittivity and permeability at these resonance frequencies.

Lorentz model is utilized for material characterization, which utilizes the information related to electron motion such as acceleration, damping force, and restoring force associated with it to relate to the electric field. The interrelation among these parameters can be presented in the Lorentz model using the mathematical form presented in Ref.^29,45^, As discussed in Ref^29^, electrical susceptibility can be determined from the solution of this equation of the Lorentz model. From this electrical susceptibility, permittivity can be determined. Similarly, using a magnetic counterpart instead of the electrical one, such as a magnetic field, the magnetic polarization Lorentz model for magnetic property can be developed and a solution of it provides magnetic susceptibility. This magnetic susceptibility helps to determine the permeability of the material. By neglecting the restoring force, from the Lorentz model Drude model is introduced. In CST, iterative simulation is performed to obtain the S_11_ and S_21_. In this method, S-parameter values are so optimized that the obtained results comply with the values of the Drude-Lorentz model and reproduce the behavior of the MTM cell^29,46^. Now, the permeability of the MTM cell has been determined using two different methods: (a) using Eq. (2) namely the NRW method, (b) using the CST post-processing module that explores the robust retrieval method. In both methods same S-parameter values obtained from the simulation in CST are used so that permeability can be obtained obeying the principle of the Drude–Lorentz model. The resulting outcomes are presented in graphical form in Fig. 13c. As depicted in Fig. 13c, both methods of permeability extraction provide the same real values for permeability at the resonance frequencies of S_21_. The real and imaginary part of the permeability follows the same changing patterns without any frequency shifting. A little deviation in maximum values of real parts and minimum values of imaginary parts between these two methods are observed. This is due to the fact that the two methods consider two different equations as solutions for obtaining the permeability. Despite this dissimilarity, both method ensures negative permeability at the frequencies of interest.

The normalized impedance, Z can be determined using the mathematical relation, Z = $\sqrt{{\upmu }_{\text{r}}/{\upvarepsilon }_{\text{r}}}$. The calculated Z values are then plotted in Fig. 13d and the near-zero values are presented in Table 4. At 3.424 GHz, 10 GHz, 14.816 GHz where the resonances occurred the extracted normalized impedances are 0.000965 + j0.0047, 0.000596 + j0.0064, 0.00314 + j0.0086 and 0.000524 + j0.00918, respectively. It is a noticeable fact that the normalized impedance has far deviated from the value 1 + j0 at every frequency of resonances of S_21_. The normalized impedance 1 + j0 indicates that the impedance of metamaterial will be essentially equal to the free space impedance. That happens when permittivity and permeability both are equal. But, in case to make the permittivity and permeability equal, the S_11_ and S_21_ need to be zero as per the Eqs. (1) and (2). That is the condition of the perfect absorber, in which all the signal will be absorbed and no signal will be transmitted or reflected from it. For the omission of the transmission in the absorber, full metallic backplane needs to be employed on one side of the substrate and on the opposite side, the resonating patch is utilized for making the reflection coefficient near zero at the targeted frequencies. Thus, permittivity and permeability become nearly equal and Z becomes closer to 1 + j0. Since the present work contains only the resonating patch over the substrate and no metallic backplane is used, a portion of the incident wave is transmitted and the rest is reflected back. Therefore, both S_11_ and S_21_ never become zero simultaneously. Thus, permeability and permittivity become unequal resulting in normalized impedance deviating from the value Z = 1 + j0. As expressed in Fig. 13b,c the scale of permittivity is higher than the permeability. The metamaterial exhibits high electric field distribution as depicted in Fig. 5. The maximum value of the electrical field is around 10,000 V/m in the region where electrical dipoles are formed. Unlikely, the magnetic field distribution presented in Fig. 6 reveals that maximum field strength is around 50 A/m. The electric dipole formed by the high electric field causes strong electrical resonances. On the other hand, the magnetic resonance is not so strong due to the comparatively lower strength magnetic dipole. Thus, permittivity is higher in scale than the permeability at the frequency of resonances. The refractive index of the proposed MTM is presented in Fig. 13e and the ranges of negative refractive index are presented in Table 4.

### Effects of substrate change of the designed MTM

**Figure 14:**
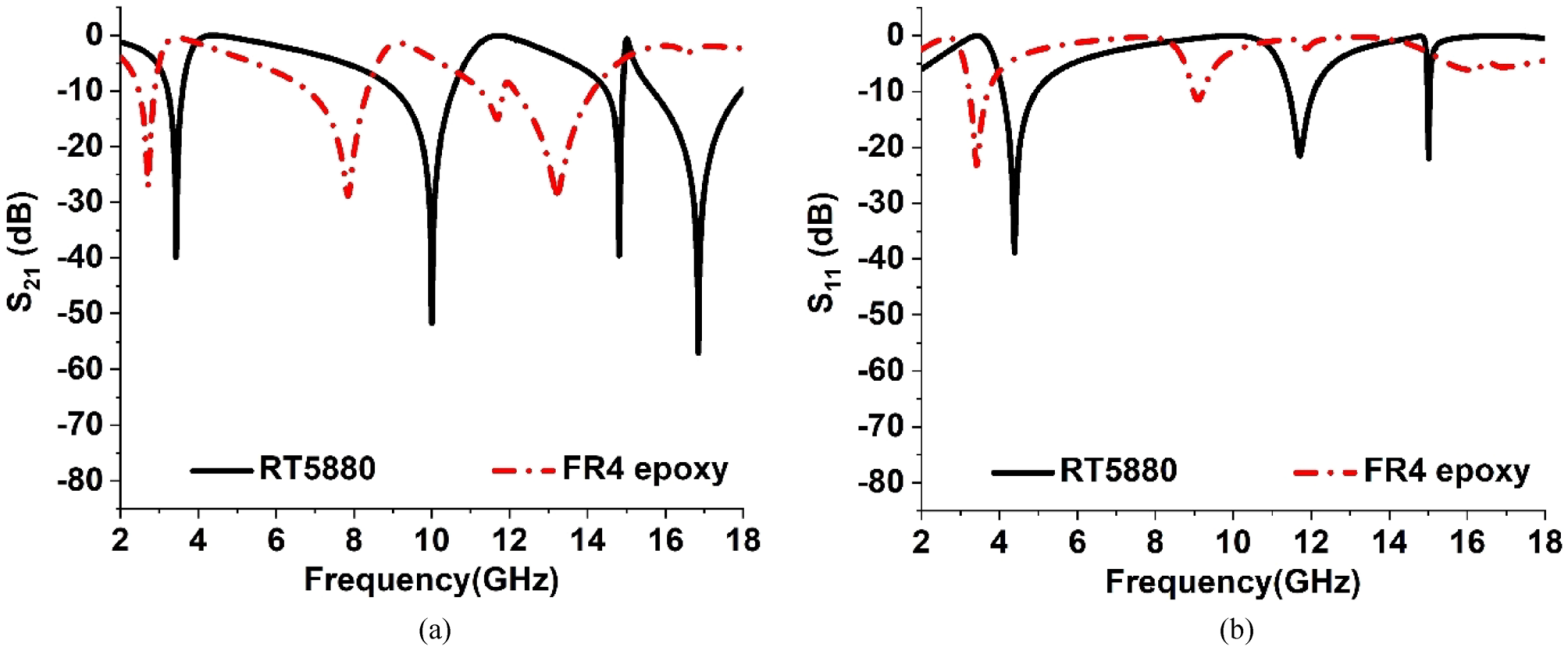
(**a**) S_21_, and (**b**) S_11_ spectra for RT5880, and FR4 epoxy substrates.

In the proposed model of MTM, Rogers (RT5880) is used as the substrate material. However, to analyze the impact of substrate change on the transmission and reflection coefficients, the simulation is also conducted for the FR4 epoxy substrate of the designed MTM. The S_21_ and S_11_ spectra for both types of substrates are presented in Fig. 14a,b respectively. For Rogers (RT5880) substrate, the resonances of S_21_ occurred at 3.424 GHz, 10 GHz, 14.816 GHz, and 16.848 GHz with peak values of − 39.86 dB, − 51.65 dB, − 39.58 dB, and − 57 dB respectively. Whereas for the FR4 epoxy substrate, the resonance of S_21_ occurred at 2.7 GHz, 7.84 GHz, 11.68 GHz, and 13.32 GHz with magnitudes of − 27.07 dB, − 28.86 dB, − 15.07 dB, and − 28.37 dB respectively. The resonances of S_11_ occurred at 4.385 GHz, 11.712 GHz, and 15.008 GHz with peak values of − 38.84 dB, − 21.52 dB, and − 22 dB respectively for our proposed MTM. The resonances of S_11_ using FR4 epoxy substrate are attained at 3.424 GHz and 9.08 GHz with peak values of − 23.14 dB, and − 11.51 dB respectively. Because of using FR4 epoxy instead of RT5880 substrate the resonances of both S_21_ and S_11_ are shifted towards the lower frequencies as shown in Fig. 14a,b respectively. In addition, the peak values of both S_21_ and S_11_ both decreased significantly for using FR4 epoxy substrate.

Due to the use of RT5880 as the substrate in the proposed MTM, we can cover the frequencies of S, X, and Ku bands. Whereas if we use FR4 epoxy as the substrate in the designed MTM, the frequency bands will shift to S, C, and X bands.

### Comparison of the unit cell of the proposed MTM with its different arrays

**Figure 15:**
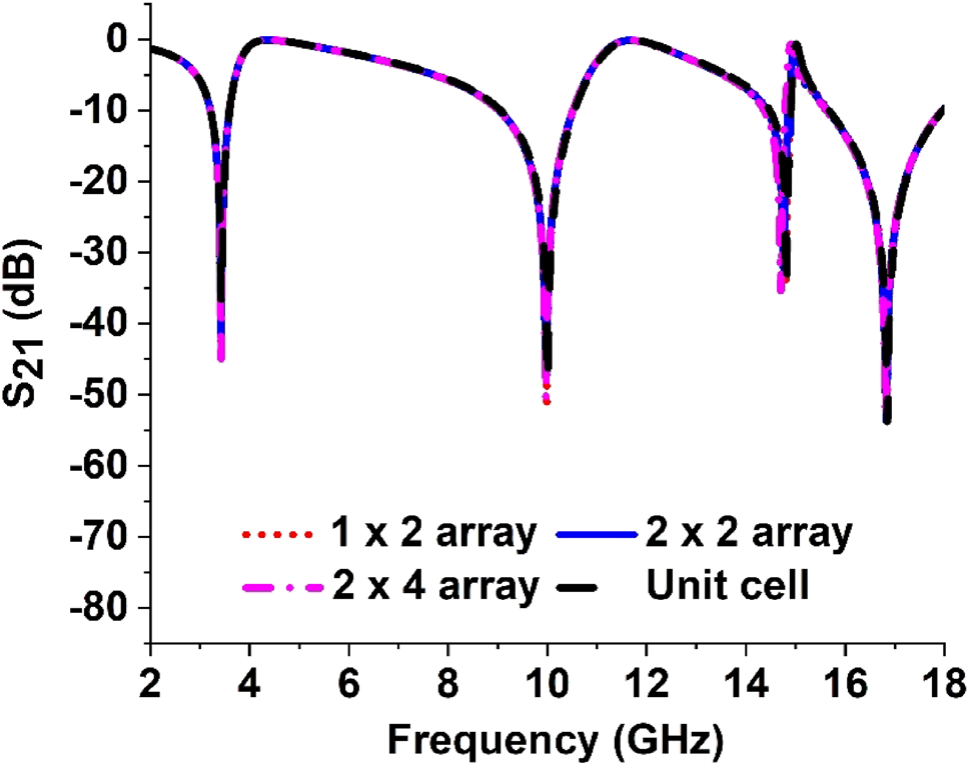
S_21_ for unit cell and different array of the proposed MTM.

The arrays of unit cells of 1 × 2, 2 × 2, and 2 × 4 are compared with the performance of the unit cell as the array arrangements are used frequently instead of the single cell in most of the practical applications of MTM. The transmission coefficients (S_21_) of these array arrangements are plotted in Fig. 15. As compared to the unit cell, the arrays of 1 × 2, 2 × 2, and 2 × 4 have no frequency shifting for first, second, and fourth resonances. Though they have a little higher magnitude increment compared to the unit cell of the proposed MTM. However, at the third resonance frequency, the 2 × 4 array has a small shift towards the left side with a higher magnitude as shown in Fig. 15. The reason behind this small mismatch is the coupling effect between the unit cells. As the variation is very small compared to the response of the unit cell no additional mechanism is considered to reduce the mismatch.

### Impact of oblique incident angle change of the proposed MTM

**Figure 16:**
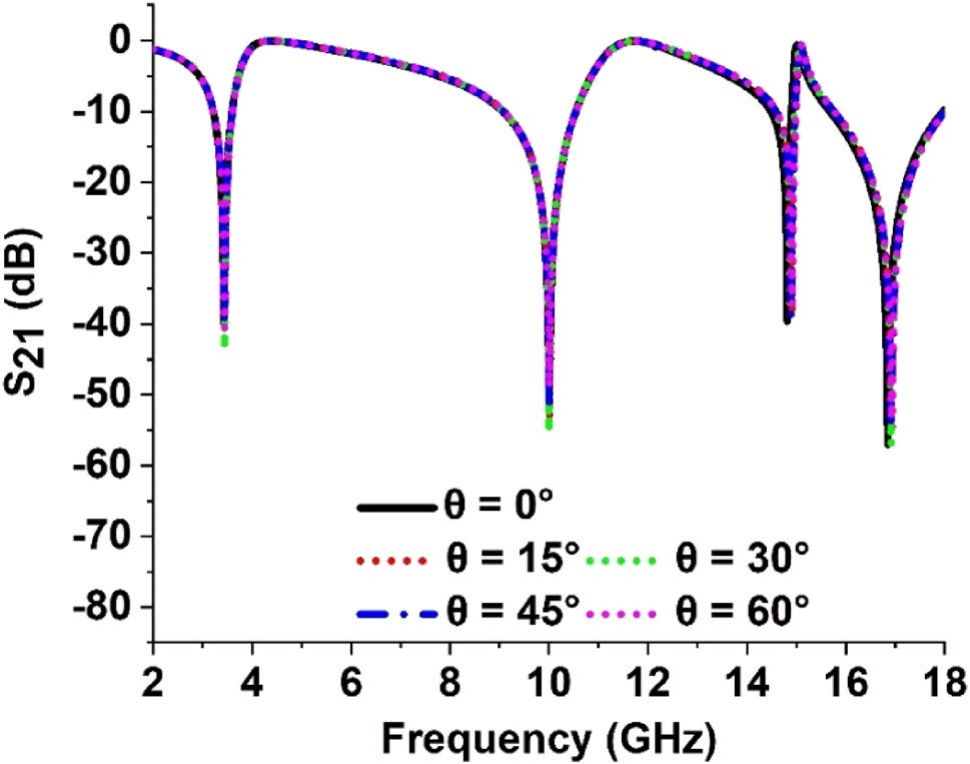
Transmission coefficient (S_21_) for different incident angle of the proposed MTM.

Roy et al*.*^47^ observed the polarization-insensitive character of the design metamaterial absorber by changing the oblique incident angle. A similar technique is adopted to observe the impact of the oblique incident angle on the S_21_ of the proposed MTM. The S_21_ spectra for incident angle (θ) change from 0° to 60° with constant increment of 15° and responses are plotted in Fig. 16. The fequencies are not changed due to the variation of incident angle however a very small variation is observed in magnitude due to this change. So the overall response of the transmission coefficient is unaltered and the proposed MTM exhibited the insensitive characteristic for incident angle. The reason behind this behavior is the mirror-symmetric structure of the MTM about the vertical axis.

### Effects of H-shaped modifiers in different positions of the unit cell

**Figure 17:**
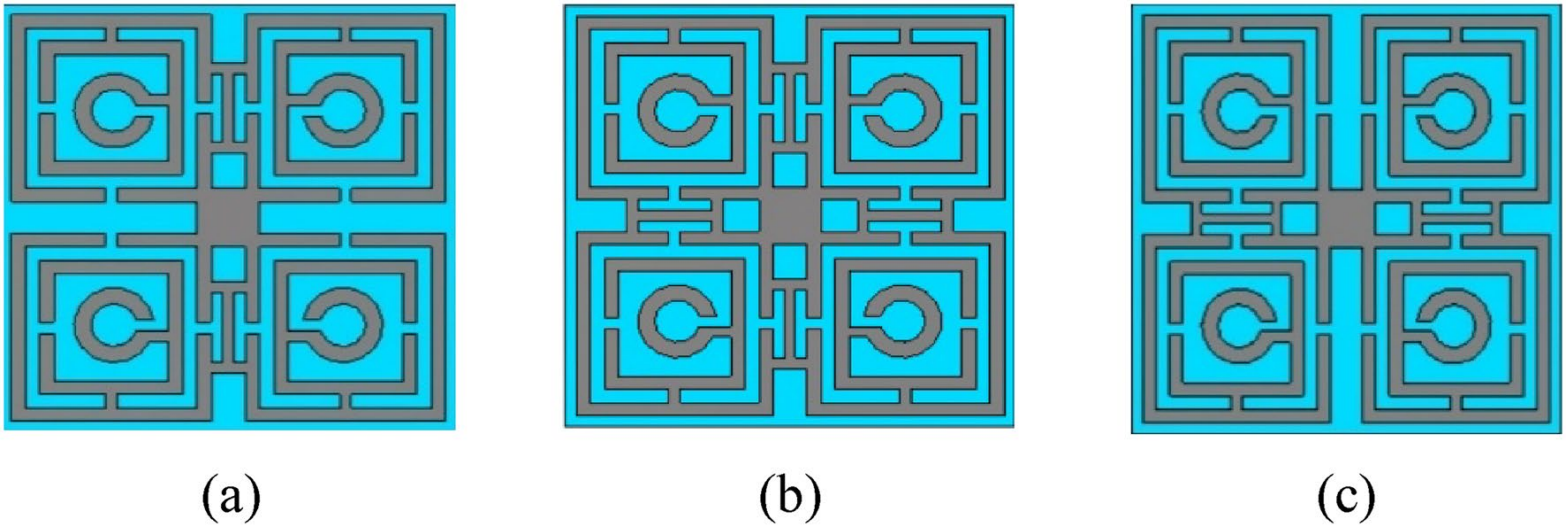
Front view of different models with H-shaped modifiers. (**a**) Model 1, (**b**) Model 2, and (**c**) Proposed Model.

**Figure 18:**
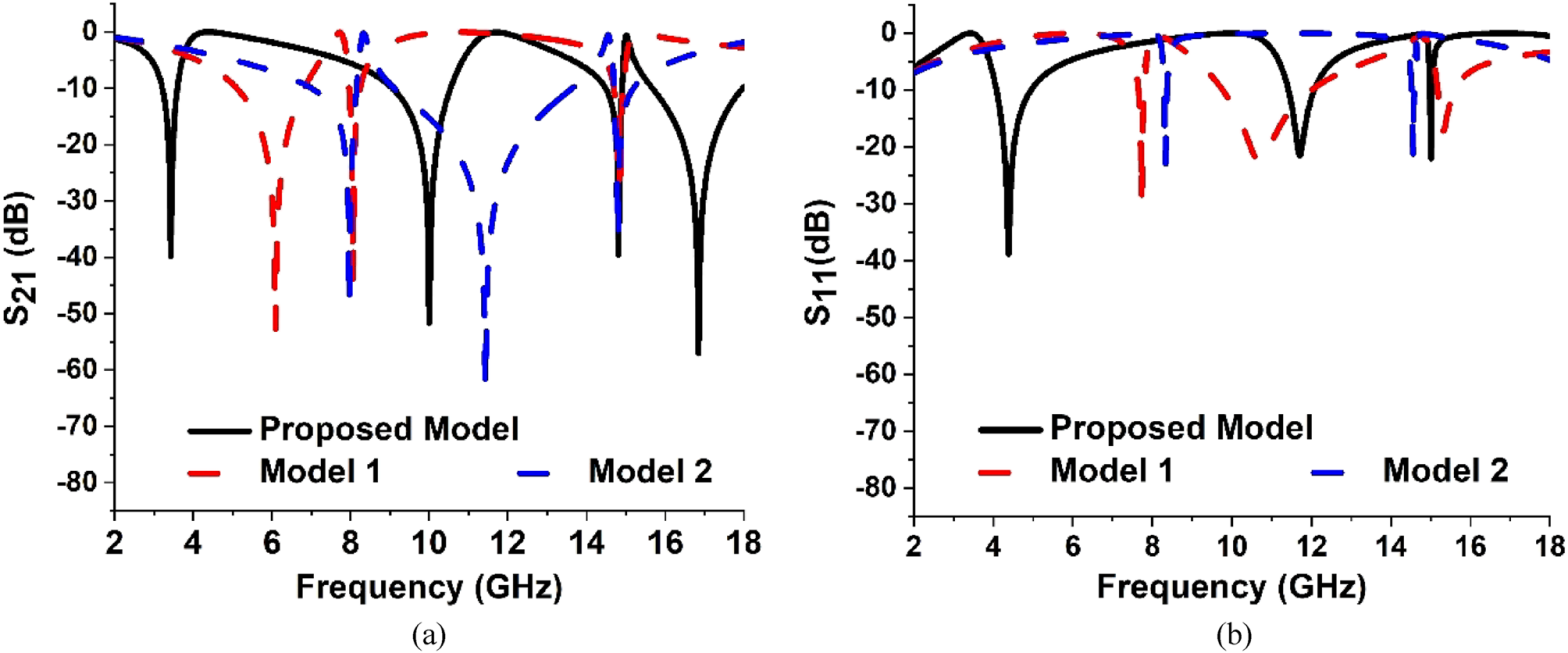
Scattering parameters: (**a**) S_21_, and (**b**) S_11_ for different positions of H-shaped modifiers in the unit cell.

The proposed model of the system is compared with two other modifications of the model in this part. These modifications are considered Model 1 and Model 2 which are given in Fig. 17a,b respectively. Model 1 is developed by inserting a conducting H-shaped converter on top of the substrate between two upper quartiles of the cell and in a similar way another H-shaped converter is placed between two bottom quartiles of the cell. These two H-shaped converters are aligned along the vertical axis of the unit cell. Model 2 is modified from Model 1, where another pair of H-shaped converters are placed along the horizontal axis of the unit cell as shown in Fig. 17c. The parameters S_21_ and S_11_ of these two models are compared with the S_21_ and S_11_ of the proposed model as presented in Fig. 18a,b respectively. The resonance frequencies and bandwidths of S_21_ and S_11_ of these two models and the proposed unit cell are specified in Tables 5 and 6 respectively.

**Table 5:** Resonance frequencies and bandwidths of S_21_ for different Models of the unit cell.

Design	Resonance frequency (GHz)	Bandwidth (GHz)
Model 1	6.10, 8.08, and 14.89	1.713, 0.2659 and 0.236
Model 2	7.98, 11.45 and 14.79	1.1297, 5.514 and 0.517
Proposed MTM	3.424, 10, 14.816 and 16.848	0.374, 1.468, 0.365 and 2.288

**Table 6:** Resonance frequencies and bandwidths of S_11_ for different models of the unit cell.

Design	Resonance frequency (GHz)	Bandwidth (GHz)
Model 1	7.77, 10.73 and 15.36	0.1454, 2.29 and 0.432
Model 2	8.337 and 14.56	0.076, 0.065
Proposed MTM	4.385, 11.712 and 15.008	0.8677, 0.629 and 0.055

Using Model 1, the three resonances of S_21_ are achieved. A significantly wide bandwidth of 1.713 GHz is attained at 6.10 GHz as shown in Fig. 18a. For Model 2, the first and second resonances have a substantial amount of shifting. A wide bandwidth of 5.514 GHz is achieved at 11.45 GHz using this model. Using the proposed model four resonances can be achieved from 3.424 to 16.866 GHz. Through this model, a wide bandwidth of 1.468 GHz and 2.288 GHz can be achieved at 10 GHz and 16.848 GHz respectively. Similarly, from Fig. 18b and Table 6, it is observed that using Model 1, three resonances of S_11_ can be achieved, where a wide bandwidth of 2.29 GHz is noted at 10.73 GHz. Using Model 2 two resonance frequencies of S_11_ and using the proposed model three resonances of S_11_ can be achieved respectively.

So using the H-shaped converters in different portions of the unit cell the frequency-switching phenomena can be observed significantly. We can switch seven different resonance frequencies of S_21_ from 3.424 to 16.848 GHz and eight different frequencies of S_11_ from 4.385 to 15.36 GHz using these frequency switching models.

### Far-field analysis of the proposed MTM

**Figure 19:**
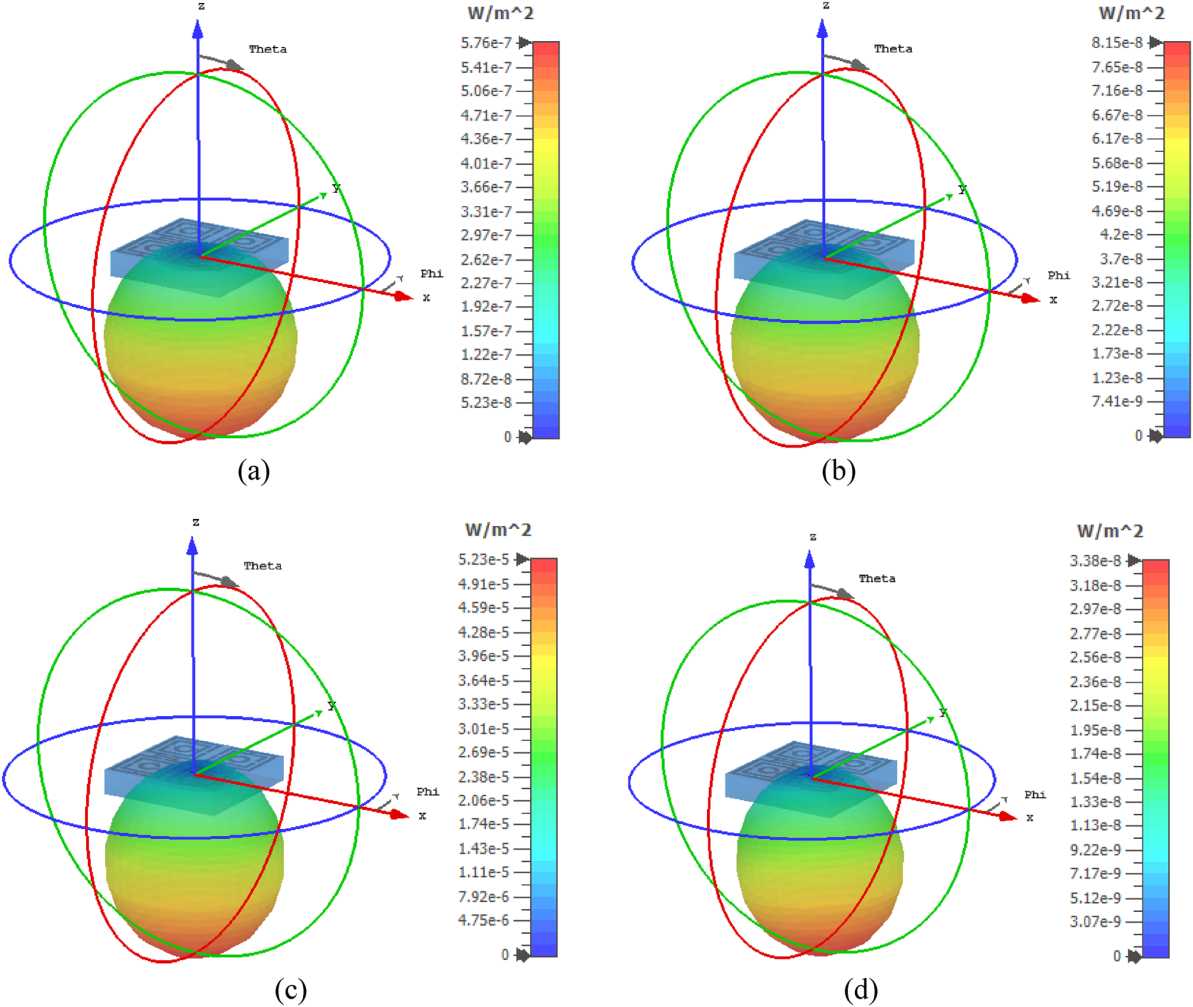
Far-field power distribution pattern of the proposed MTM for: (**a**) 3.424 GHz, (**b**) 10 GHz, (**c**) 14.816 GHz, and (**d**) 16.848 GHz.

The far-field characteristics of the MTM are studied considering the effect of the structure when the incident electromagnetic wave is imposed on the resonator from the + z direction. The far-field power distribution pattern is presented in Fig. 19a–d for the four frequencies of interest for which transmission resonances are obtained. Figure 19a illustrates the power density for 3.424 GHz which provides the maximum radiated power is 5.757 × 10^−7^ W/m^2^. Moreover, at this frequency, the radiation efficiency of the MTM is only 0.0165. At 10 GHz, the maximum obtained power is 8.15 × 10^−8^ W/m^2^ and radiation efficiency is 0.0006. The power distribution pattern for this frequency is expressed in Fig. 19b. As illustrated in Fig. 19c, at 14.816 GHz, the pattern becomes more concentric toward the − z direction compared to the earlier two distribution patterns. It exhibits a low amount of power with a maximum value of 5.228 × 10^−5^ W/m^2^ and the radiation efficiency is 0.01247. The directivity further increases at 16.848 GHz as shown in Fig. 19d where the maximum power is 3.38 × 10^−8^ W/m^2^ and radiation efficiency is 0.0001. The far-field radiative power analysis indicates that almost zero electromagnetic radiative power is emitted from the MTM with very negligible efficiency (nearly zero). Thus, the MTM does not act as a radiator. Moreover, transmission coefficients at the frequency of interest are nearly zero which provides the reflection coefficients of 0.998, 0.998, 0.986, and 0.998 at 3.424 GHz, 10 GHz, 14.816 GHz, and 16.848 GHz respectively. Thus, the MTM acts as a good reflector at the discussed frequencies of interest and also as a good stop-band filter for the omission of transmitted signals at these frequencies.

### Comparison of proposed model with relevant works

A comparative analysis is presented in this section with the help of Table 7 where the proposed model is compared with other relevant research works on metamaterials. The comparison is made based on the dimension, substrate material, resonance frequency, effective medium ratio (EMR), and covering frequency bands. The EMR is calculated using the formula, $\text{EMR}=\frac{\lambda }{l}$ where wavelength λ is considered at the lowest resonance frequency and *l* is the longer dimension of the proposed MTM unit cell. The EMR value of the MTM represents the compactness of the unit cell. High EMR indicates more compactness of The MTM. The consideration of EMR in MTM design is very important from the perspective of miniaturization of the device where the MTM can be implemented. Small-dimensioned MTM (having high EMR) can be used with the miniaturized antenna to improve the gain and directivity. Thus, EMR exhibits its impact on MTM as well as on the antenna. Refs.^36,48–51^ mentioned in Table 7 used FR4 material as the substrate of the unit cell. FR4 is the lossy medium, and MTM constructing with FR4 substrate suffers from increased energy loss, especially when the frequency is over 10 GHz. However, in our proposed model, Rogers (RT5880) is used which can minimize this limitation and can be more suitable for high-frequency applications. On the other hand, Refs.^36,48,50,52^ have larger dimensions than the proposed MTM. Moreover, the proposed MTM exhibits a higher EMR of 10.95 than the MTMs of^36,49,52^, which indicates the superiority of the proposed design in terms of compactness over those MTMs. Additionally, The MTMs of Refs.^48,49^ can exhibit resonance phenomena at a single frequency of 2 GHz and 7.435 respectively, whereas our proposed MTM can work at multiple frequency bands like the MTMs of Refs.^36,50–52^. Thus, the proposed MTM can be a good candidate as a component of high frequency application devices due to its compact dimension, multi-frequency responses compared to other MTMs.

**Table 7:** Comparison of the proposed model with other reported works.

Referred relevant articles	Dimension of the unit cell	Substrate material	Resonance frequencies (GHz)	EMR	Coveting frequency bands
^36^	9 mm × 9 mm	FR4	4.15, 10.84, 14.93	8.03	C, X and Ku bands
^48^	50 mm × 105 mm	FR-4	2	Not specified	L-band
^49^	5.5 mm × 5.5 mm	FR4	7.435	7.33	C band
^50^	11 mm × 11 mm	FR4	1.54, 3.5, and 10.4	17.7	L, S and X bands
^51^	6 mm × 6 mm	FR-4 epoxy	4.32, 7.55, and 9.76	11.57	C, and X bands
^52^	20 mm × 20 mm	RT5880	3.67, 4.74, 8.38, and 10.8	7.57	L, S, C, and X bands
Proposed Model	8 mm × 8 mm	RT5880	3.424,10, 14.816, and 16.848	10.95	S, X, and Ku bands

## Conclusion

In this paper, an H-shaped modifier loaded mirror-symmetric metamaterial is presented for multi-band wireless communication. The proposed model exhibits negative permittivity, permeability, and refractive index. The EMR of the model is found 10.95, which indicates the compactness of it. The resonances of S_21_ are found at 3.424 GHz, 10 GHz, 14.816 GHz, and 16.848 GHz. The simulated outcome is compared with the measurement results of the prototype using VNA where the frequency deviation found was very small. This indicates that the simulation result is well matched to the measurement result. The resonance phenomena is further studied through electro-magnetic field and surface current analysis, and equivalent circuit modeling. Along with the proposed model, two other versions of H-shaped modifier-loaded metamaterial were also developed named Model-1 and Model-2. From the comparative analysis, it is found that between 6 and 15 GHz, these two models can also cover six different resonance frequencies. Because of the compact size, negative permittivity, and permeability of the proposed metamaterial, it can be utilized to improve the performances of microwave communication elements such as implementation as a part in antenna, sensor, and band-stop filter. As the proposed MTM can cover S, X, and Ku bands it can be implemented in 5G devices, satellites for weather monitoring systems, and dish antennas for VSAT to improve their performances.

## Data Availability

The datasets generated and analysed during the current research work are not publicly available due to the restriction of Universiti Kebangsaan Malaysia (affiliated institution) but are available from the corresponding author on reasonable request for further progress of the research work.
